# Understanding Alzheimer's disease as a disorder of consciousness

**DOI:** 10.1002/trc2.12203

**Published:** 2021-11-29

**Authors:** Jonathan D. Huntley, Stephen M. Fleming, Daniel C. Mograbi, Daniel Bor, Lorina Naci, Adrian M. Owen, Robert Howard

**Affiliations:** ^1^ Division of Psychiatry University College London London UK; ^2^ Wellcome Centre for Human Neuroimaging University College London London UK; ^3^ Department of Experimental Psychology University College London London UK; ^4^ Max Planck‐UCL Centre for Computational Psychiatry and Ageing Research University College London London UK; ^5^ Department of Psychology Pontifical Catholic University of Rio de Janeiro Rio de Janeiro Brazil; ^6^ Institute of Psychiatry, Psychology & Neuroscience King's College London London UK; ^7^ Department of Psychology University of Cambridge Cambridge UK; ^8^ School of Psychology Global Brain Health Institute Trinity College Dublin Dublin Ireland; ^9^ Brain and Mind Institute Department of Physiology and Pharmacology and Department of Psychology University of Western Ontario London Ontario Canada

**Keywords:** Alzheimer's disease, awareness, consciousness

## Abstract

People with Alzheimer's disease (AD) demonstrate a range of alterations in consciousness. Changes in awareness of cognitive deficit, self‐awareness, and introspection are seen early in AD, and dysfunction of awareness and arousal progresses with increasing disease severity. However, heterogeneity of deficits between individuals and a lack of empirical studies in people with severe dementia highlight the importance of identifying and applying biomarkers of awareness in AD. Impairments of awareness in AD are associated with neuropathology in regions that overlap with proposed neural correlates of consciousness. Recent developments in consciousness science provide theoretical frameworks and experimental approaches to help further understand the conscious experience of people with AD. Recognition of AD as a disorder of consciousness is overdue, and important to both understand the lived experience of people with AD and to improve care.

## 1 INTRODUCTION

Alzheimer's disease (AD) is a major cognitive disorder, leading to progressive impairment in cognition, function, and a range of behavioral and psychological symptoms.^1^ What has traditionally and typically been missed from descriptions of AD, however, are the effects on conscious experience.

**RESEARCH IN CONTEXT**

1. Systematic review: This paper reviews evidence that Alzheimer's disease (AD) involves disruption of components of awareness and arousal. Deficits in higher‐level awareness occur in mild AD, with lower levels of awareness often preserved into the severe stages. However, there is significant heterogeneity among patients and a pressing need to identify objective and reliable biomarkers of awareness in AD to understand the range of alterations in conscious experience and appropriately enhance the content and quality of person‐centered care.
2. Interpretation: Patterns of neurodegeneration in brain networks seen in AD correspond to observed dysfunction in awareness and arousal. Behavioral and neurobiological evidence from AD reciprocally informs current theories of consciousness and awareness and can facilitate investigation of neural correlates of consciousness.
3. Future directions: We outline how biomarkers developed to assess consciousness in different patient groups could identify awareness in AD, including at the severe stages of the disease, and how understanding AD as a disorder of consciousness could improve clinical care and help caregivers.

A widely used categorization of consciousness includes two major components: arousal (i.e., the state of consciousness) and awareness of the self and the environment (i.e., the contents of conscious experience; see Box 1: Glossary).^2^ Diagnostic criteria include impairment of awareness as a key item for delirium, but not dementia, and “disorders of consciousness” usually refer to disorders affecting arousal, such as coma, rather than AD.^1^ This underlines a lack of understanding of consciousness and its centrality to the lived experience and deficits of people with AD. For example, the most distressing aspect of AD for patients and caregivers is not loss of memory per se, but the change in subjective experience and awareness of themselves, of others, and their external environment that accompanies the condition.^3^ For caregivers of someone with advanced AD, there is uncertainty as to what the person with AD is now able to experience, and feelings of grief for the loss of the person they knew, described as being akin to a premature bereavement.^4^ Understanding the subjective experience of people with severe AD is central to the ambition of person‐centered care and to maximizing quality of life.^3^

**BOX 1 GLOSSARY**

**CONSCIOUSNESS—**refers to the state of wakefulness or arousal, (e.g., coma vs. awake) and awareness, the phenomenal experience of being conscious.

**OBJECTS OF AWARENESS—**refers to subjective experiences of being aware of an object or objects. These objects could be external stimuli or events or internal states (including mental representations, aspects of the self, emotional states).

**ANOSOGNOSIA—**describes the lack of awareness of deficit in the context of disease.

**METACOGNITION—**refers to knowledge, monitoring, and regulation of one's own cognitive processes (i.e., “thinking about one's own thinking”). This can be considered “locally” where one is monitoring and appraising current or recent cognitive performance in specific cognitive domains (e.g., metamemory). It may also be considered “globally” where an individual reflects on their overall cognitive or functional abilities in a more global sense, which may include elements of reflection on the self and making comparisons against previous abilities, autobiographical memories, or expected standards.

**AUTONOETIC CONSCIOUSNESS—**refers to the “higher level” subjective conscious experience that accompanies episodic memory retrieval, which is often associated with a sense of “mental time travel” back to the event and may encompass emotional feeling, autobiographical knowledge, and continuity of the self.

**NOETIC CONSCIOUSNESS—**refers to the subjective experience of “knowing” that may accompany semantic memory but is a less phenomenologically rich subjective experience than autonoetic consciousness, and does not contain the same higher awareness of self or “mental time travel”.

**SELF‐AWARENESS—**refers to the constellation of subjective experiences that give rise to a sense of self. At its simplest it refers to the capacity to experience the physical body as associated with the self and to separate internally from externally derived stimuli. At a higher level it involves the ability to reflect on the self as the object of awareness, which includes access to autobiographical knowledge and memories, beliefs and attributes, wider social identity, self‐evaluation, and “meta‐self‐awareness” (being aware that one is oneself self‐aware).

**INTROSPECTION—**refers to intentional self‐monitoring and evaluation of ongoing conscious experiences, when the object of awareness is one's own conscious thoughts.

**REALITY MONITORING—**refers to the ability to correctly attribute the origins of information subjectively experienced as either arising from internal cognitive functions such as thought and imagination, or from the outside world.

Despite these clear clinical and ethical imperatives, the subject of consciousness has remained peripheral to clinical criteria for AD. Our argument is that dysfunction of aspects of consciousness is a central phenomenological characteristic of AD, becoming more pronounced as the disease progresses. This has been hiding in plain sight, as cognitive, functional, and behavioral impairments have traditionally been the clinical focus, while a central unifying issue of change in conscious awareness, which is of great importance to patients and families, has generally not been recognized or articulated. We suggest that AD leads to dysfunction of components of awareness and arousal, related to the underlying progression of neurodegeneration. We observe that, although the general trajectory may be of progressive dysfunction, there is significant heterogeneity and some components of awareness of self and the environment may persist into the advanced stages of the disease. This highlights the need to identify objective and reliable biomarkers of awareness, to further understand AD as a disorder of consciousness and the central importance of person‐centered care. Behavioral and neurobiological evidence from AD reciprocally supports current theories of consciousness and can facilitate investigation of neural correlates of consciousness (NCC). We outline how biomarkers developed to assess consciousness in other patient groups could identify awareness in AD, and how understanding AD as a disorder of consciousness will improve clinical care and help caregivers.

## 2 AD LEADS TO DYSFUNCTION OF AWARENESS AND AROUSAL

**FIGURE 1:**
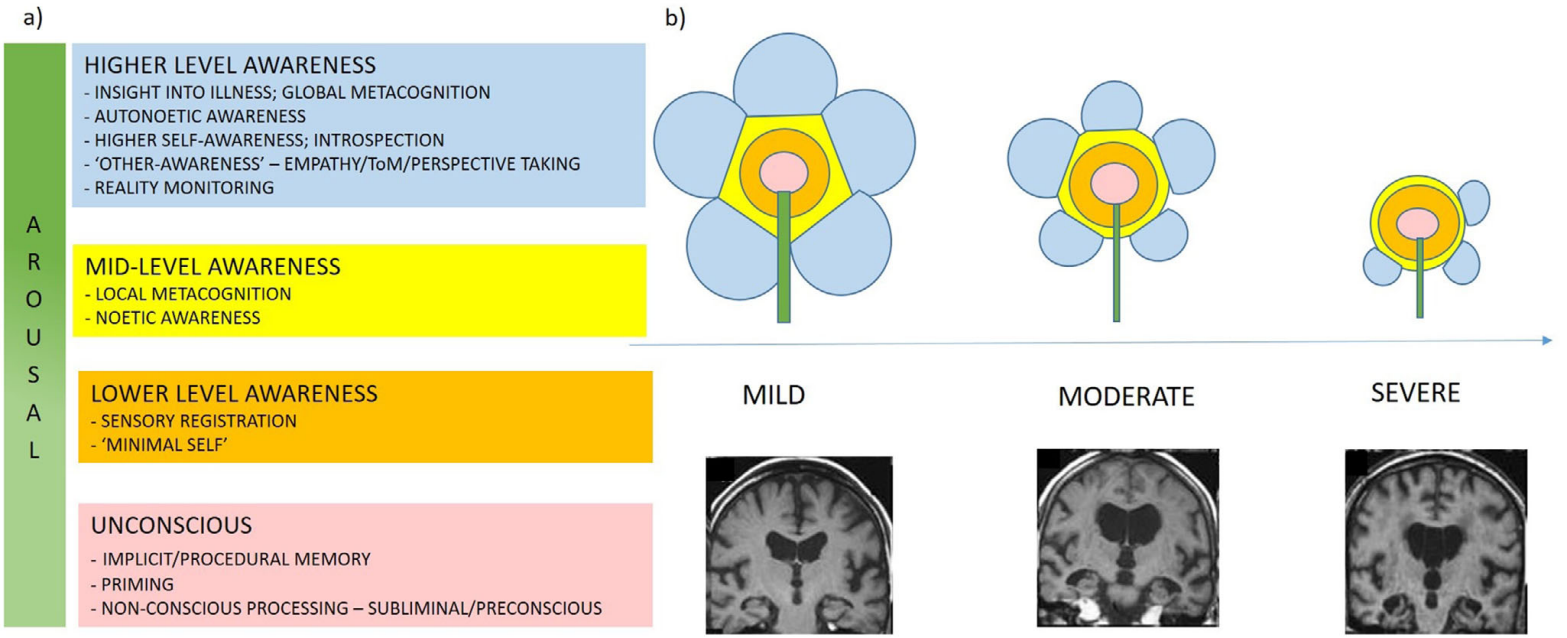
A) The potential hierarchical structure of some components of awareness and arousal. B) A conceptual illustration of the potential deterioration in different levels and facets of awareness and arousal with the progression of Alzheimer's disease (AD; colors correspond with categories in [A]). Behavioral evidence suggests components of higher awareness become impaired in mild AD, and awareness generally contracts with disease progression. However, the pattern of impairment is heterogeneous and some aspects of higher awareness may persist in some individuals. Magnetic resonance images of mild (Scheltens 2), moderate (Scheltens 3), and severe AD (Scheltens 4) adapted from Wahlund et al.^8^

Bringing together different models of awareness^5^, ^6^ with clinical observations of people at different stages of AD would build a picture of how AD affects components of awareness and arousal, and how these change with disease progression. Current models of the components of conscious awareness suggest there are a number of potential objects of awareness (see Box 1: Glossary), which can either be external stimuli, or internal mental, emotional, or physical states.^5^ The objects or contents of awareness can be experienced on multiple levels, from higher levels of awareness (e.g., the rich integrated experience of day dreaming, for example, about the feeling of lying on a beach on holiday), to lower‐level minimal awareness of the self and sensory registration (e.g., simple sensory awareness of feeling hot with no access to context or self‐reflection).^6^ Figure 1 illustrates potential hierarchical components of awareness of the self, others, and the environment (Fig 1a), and how these may change with the progression of AD (Fig 1b). While these have been examined in isolated studies of people with AD, most studies have only assessed people at the mild (e.g., Clinical Dementia Rating Scale [CDR]^7^ stage 1) or moderate (e.g., CDR 2) stages of the disease. There is a striking lack of empirical research in people at the more severe stages (e.g., CDR 3), who may be unable to communicate their subjective experiences, and a coherent picture of how AD progression impairs consciousness is lacking. The following components of awareness are by no means an exhaustive list, and overlap conceptually and neurobiologically. However, they provide a useful framework to describe AD as a disorder of consciousness.

### 2.1 Higher‐level awareness

Higher‐level awareness consists of a variety of components. Impaired awareness of the presence or degree of general cognitive and functional deficits, or of the diagnosis itself, often referred to as “anosognosia,” is seen in up to 80% of people with AD.^9^ Studies generally suggest that awareness at this level becomes increasingly impaired with progression of AD; however, some individuals with severe AD may still show some limited awareness of their cognitive decline.^9^, ^10^, ^11^, ^12^, ^13^ Anosognosia is related to other overlapping features of higher‐level awareness and cognitive function affected by AD, and to the integration of these processes (see Box 2). A component of higher‐level awareness associated with episodic memory is autonoetic consciousness. This was originally defined by Tulving as “the kind of consciousness that mediates an individual's awareness of their existence and identity in subjective time,”^14^ and there is evidence that this higher‐level component of awareness is impaired In mild–moderate AD.^15^, ^16^, ^17^, ^18^, ^19^ Autonoetic consciousness is intrinsically related to self‐awareness^6^, ^20^ (see Box 1: Glossary). While more abstract self‐awareness also becomes impaired in mild AD, alongside anosognosia,^21^, ^22^, ^23^, ^24^, ^25^ a degree of semantic knowledge of the self may be preserved.^26^ This enables some continuity of the awareness of self in people with mild–moderate AD, although an impaired ability to monitor and update self‐relevant knowledge may lead to reliance on older self‐knowledge, which has been referred to as the “petrified self” in AD.^27^ There is evidence for reduction in the closely related ability for introspection and reflection on personal thoughts, feelings, and beliefs, even in mild AD.^22^, ^24^ However people with mild AD may only demonstrate reduced introspection and mind‐wandering, compared to healthy controls, during cognitively demanding tasks.^26^, ^28^ There is also evidence that people with mild–moderate AD are impaired on imaginative tasks compared to healthy controls^29^ and on differentiating imagined from external events, referred to as reality monitoring.^30^, ^31^ Impaired reality monitoring may relate to the phenomena of misidentification delusions seen in AD (e.g., that a caregiver has been replaced by an imposter). These delusions are associated with perceptual distortions and the assignment of aberrant salience to environmental stimuli,^32^ but also involve impaired conscious reflection and reality monitoring. Similar processes might also contribute to a failure to evaluate and update self‐knowledge in anosognosia.^27^

**BOX 2 Consciousness and cognitive function in AD**

AD results in impairments in multiple cognitive domains, including attention, working memory, episodic memory, language, and executive functioning. There is evidence that consciousness per se can be dissociated from content‐specific sensory or cognitive processes.^33^ Some elements of higher awareness, for example deficits in complex theory of mind (ToM), are strongly associated with executive and general cognitive impairment,^34^ while others such as local metacognition can be experimentally dissociated from task performance to demonstrate specific metacognitive deficits.^35^ Autonoetic consciousness is intrinsically linked with episodic memory, and episodic autobiographical memory and semantic self‐knowledge support self‐awareness.^26^, ^36^ The Cognitive Awareness model proposes heterogeneous mechanisms for anosognosia in AD, related to these underlying cognitive deficits.^37^ Within this model, anosognosia may result from executive impairments in evaluating and monitoring performance (“executive anosognosia”) or in encoding and retrieval of up‐to‐date autobiographical episodic memory (“mnemonic anosognosia”).^37^ Similarly, attention and executive components of working memory implicated in some theories of consciousness^38^ are impaired in AD. Although in general the evidence suggests that deficits in awareness mirror disease progression and cognitive decline, the interplay between cognitive domains and conscious awareness needs further study. There appears to be no simple linear relationships among executive function, episodic memory, or general cognition and awareness, which may be an epiphenomenon of complex cognitive function.^27^ AD provides an important opportunity to assess how conscious awareness may correlate with differential impairments in executive, attentional, and episodic memory function as the disease progresses to further clarify how conscious awareness may be reliant on, or dissociable from, other cognitive processes.

An aspect of higher‐level awareness that is required for successful social cognition involves accurate recognition of emotions and awareness of the mental states of others, which in turn depend on the capacity for empathy, theory of mind (ToM), and perspective taking. In mild AD there is evidence for impairment on complex, higher‐order ToM tasks, while more basic ToM reasoning remains preserved.^39^, ^40^, ^41^ Similarly, studies in people with mild AD have found deficits in empathy and heightened automatic synchronization of emotional states with others, referred to as emotional contagion,^42^, ^43^ compared to healthy older people. Perspective taking may be preserved in people with mild AD,^44^ even in the context of impaired judgments of their own performance.^45^ People with mild^46^ and mild–moderate AD^47^ may also have impaired awareness of their own social functioning and behavior in relationships. While there is variability in higher‐level social awareness in early AD, this appears to decline as dementia progresses into the moderate–severe stages,^34^, ^48^ although inferences made from observations suggest some people with severe AD may retain some ability to empathize with others.^13^

### 2.2 Noetic awareness and local metacognition

In contrast to higher levels of autonoetic awareness, noetic consciousness encompasses the subjective experience of “knowing” based on semantic knowledge of the world in the present context.^14^ This lacks the experiences of (for example) mental time travel and higher self‐awareness that accompany autonoetic consciousness.^14^, ^20^, ^49^ There is some evidence for preserved noetic awareness in mild AD, in the context of impairment in higher autonoetic consciousness.^15^, ^16^, ^17^, ^50^

In contrast to global metacognition, which describes an ability to recall and make comparisons against previous abilities, local metacognition refers to the self‐monitoring of cognitive performance on an ongoing task in the present.^13^, ^44^ Several studies have demonstrated impaired local metacognition for episodic memory performance in mild AD,^51^, ^52^ with patients both overestimating^51^, ^53^ and underestimating their recall abilities.^54^ Some studies, however, have demonstrated preserved local metacognition on episodic memory tasks^55^ and semantic memory tasks,^51^, ^52^ including in the context of impaired global metacognition,^56^ which may reflect the different metacognitive tasks used,^51^, ^52^ and individual variation in metacognitive ability in mild AD. In people with more severe dementia, the ability to self‐recognize may require similar cognitive abilities to monitoring performance,^12^, ^57^ and some studies suggest aspects of simple self‐recognition are retained even in severe AD.^13^

### 2.3 Lower‐level awareness

At the lowest level of awareness, also referred to as the “minimal” self^58^ or sensory registration level,^12^ awareness is limited to the registration of, and basic behavioral response to, stimuli in the immediate present.^12^, ^13^, ^58^ In people with mild–moderate AD who are able to verbally communicate, such sensory experiences are straightforward to assess by self‐report and there is little doubt that this lower level of awareness remains largely intact in mild–moderate AD.^59^ However, self‐report methods are often unreliable in people with moderate–severe AD, and non‐verbal indicators have been used to assess lower levels of awareness. In a recent review, eight studies suggested that sensory registration is maintained even in people with severe AD.^13^ These studies examined a range of non‐verbal indicators of awareness including facial expression, body language, and physiological responses, evoked by a range of pleasant and unpleasant stimuli, including touch and music.^13^

### 2.4 Implicit and procedural learning

Procedural learning refers to implicit unconscious motor, perceptual, and cognitive skills, acquired primarily through practice. A review of 22 studies in mild–moderate AD, and one involving severe AD, all found evidence of preserved implicit motor‐skill learning in AD, regardless of the tasks used which included tracing, rotor pursuit, and serial reaction time.^60^ Both preserved and impaired priming effects have been found in AD,^61^ with impairment on conceptual (meaning‐based) priming tasks, but preserved priming abilities on word‐identification priming tasks in mild–moderate AD.^61^, ^62^ However, progression to severe AD limits performance of even perceptual priming tasks.^63^, ^64^ More recently, studies have suggested that implicit information processing can extend to more complex stimuli in people with mild–moderate AD, including the indirect demonstration of knowledge about cognitive deficits, despite total or partial lack of explicit acknowledgement.^65^, ^66^

### 2.5 Arousal

For any conscious experience or awareness to occur, a sufficient degree of arousal is necessary. Many clinical disorders of consciousness are also conceptualized as disorders of arousal (e.g., coma) and dysfunction in arousal may be a clinical feature of AD from the early stages.

Dysregulation of systemic circadian rhythms, specifically those involved in the sleep‐wake cycle and activity may occur in preclinical AD,^67^ are found in 25% to 40% of people with mild to moderate AD^68^ and worsen with disease progression.^69^ Dysfunction in arousal and circadian rhythms in AD may manifest as insomnia at night, increased daytime sleepiness, “sundowning” (increased agitation in the evenings), and apathy.^68^, ^69^, ^70^ Dysfunction in arousal may interact with impairments in awareness to give rise to some clinical features and phenomenology seen in AD. For example, dysregulation of arousal, in combination with impaired reality monitoring and salience mapping of socio‐emotional stimuli may contribute to agitation, anxiety, or delusions.^32^, ^71^ In the advanced stages of AD, arousal may become significantly impaired to the extent that patients with severe dementia may have reduced responsiveness, although very rarely to the extent of meeting criteria for a vegetative state.^72^ Fluctuations in arousal and lucidity have been described even in severe AD,^3^ and clarification of how these represent changes in awareness remains an important clinical question.

In summary, behavioral evidence suggests that components of higher awareness become impaired from the early stages of disease. While there is evidence that alterations in subjective conscious awareness generally progress with increasing disease severity, what is striking is the heterogeneity seen both clinically and in the literature. There are reports of elements of preserved awareness present in some individuals even at the most severe stages, despite overall trends, and this is mirrored in the reported experiences of caregivers.^3^ This highlights the need to understand how underlying neuropathological processes in AD relate to differential impairments of consciousness.

## 3 NEURODEGENERATION IN AD AND DYSFUNCTION OF CONSCIOUSNESS

**FIGURE 2:**
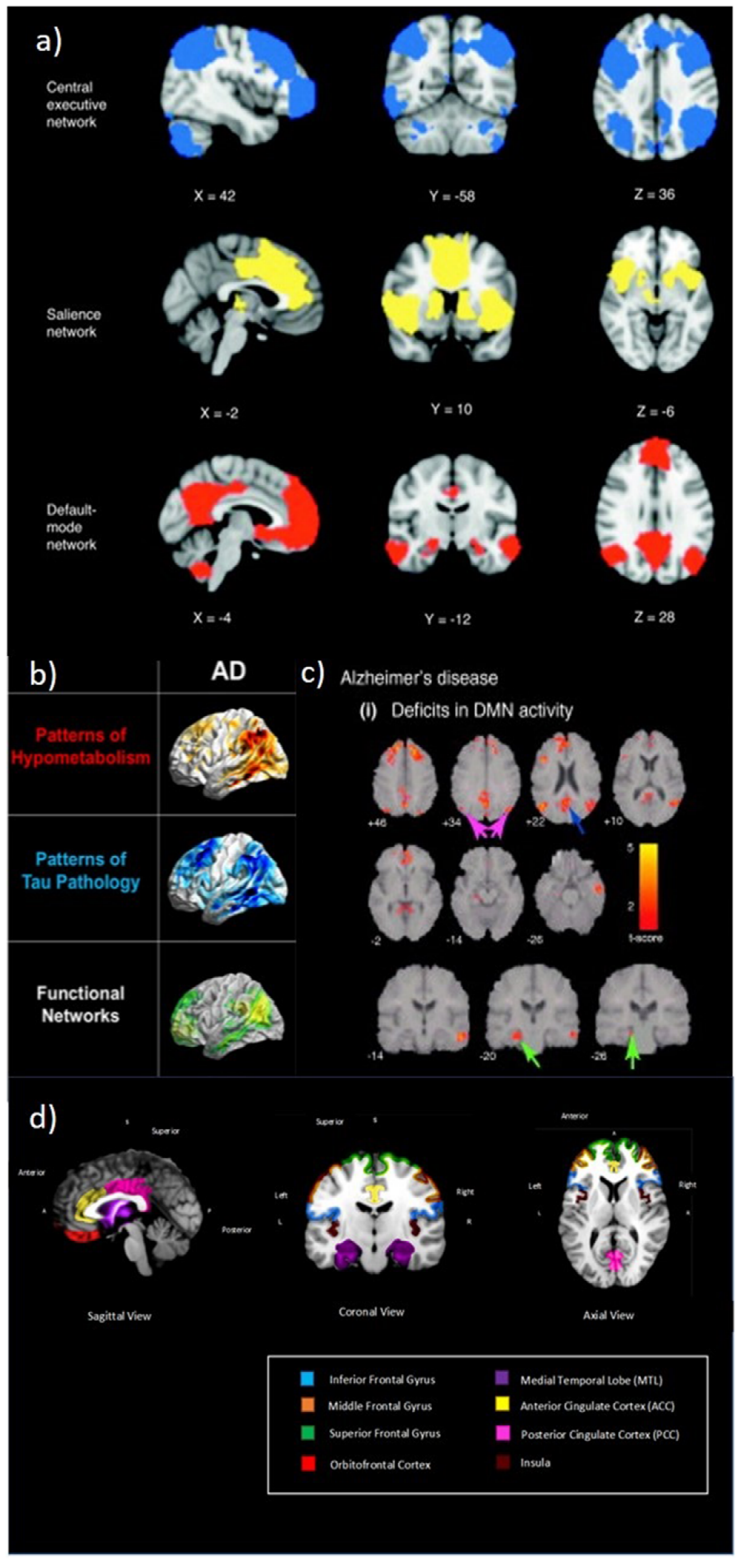
A, Principal brain regions in the central executive, salience, and default‐mode networks (adapted from Menon.^78^ B, Overlap between hypometabolism on 18F‐FDG PET, tau aggregation on 18F‐T807 PET, and default mode networks from resting‐state functional MRI in AD (adapted from Drzezga^79^). C,  Changes in DMN activity in patients with AD compared to age‐matched healthy elderly controls. The PCC (blue arrow), angular gyrus in the inferior parietal cortex (magenta arrow) and hippocampus (green arrow) show prominent activity changes in AD (adapted from Menon^78^). D, Common regions of atrophy and functional impairment associated with deficits in higher level awareness and metacognition in early AD (adapted from Hallam and Huntley^73^). AD, Alzheimer's disease; DMN, default mode network; FDG, fluorodeoxyglucose; MRI, magnetic resonance imaging; PCC, posterior cingulate cortex; PET, positron emission tomography

Despite heterogeneity in the results of studies measuring awareness at different levels, a recent review of 32 studies found key overlapping regions of the brain associated with anosognosia, autonoetic consciousness, and metacognition in early AD. These include prefrontal cortex (inferior frontal gyrus, superior frontal gyrus, medial frontal gyrus, orbitofrontal cortex), medial temporal lobe (MTL), anterior (ACC), and posterior cingulate cortex (PCC) and insula^73^ (Figure 2d). Similar brain regions and networks have been linked to deficits in imagination,^29^, ^74^, ^75^ reality monitoring,^76^ and empathy^77^ in mild AD.

Several of these areas represent nodes of the default mode network (DMN), including the medial prefrontal cortex, posterior cingulate cortex, inferior parietal lobes, and hippocampi (Fig 2a).^80^ The DMN is associated with self‐related cognitive activity, including introspection, mind‐wandering, autobiographical memory, future thinking, and social function,^78^, ^81^ as well as monitoring the external environment and shifting between contextually relevant information.^82^ Amyloid beta deposition, atrophy, and altered functional connectivity in the DMN occurs early in AD (Fig 2b and c)^58^, ^78^, ^81^, ^83^ and many of its component brain regions, such as ACC, MTL, prefrontal cortex, and insula, are considered central to theoretical models of awareness in AD.^27^, ^37^ Similarly, fronto‐parietal central executive networks (CEN, Fig 2a), have been associated with control of attention, working memory, and contents of consciousness.^38^

The salience network (SN) has nodes in the ACC and frontoinsula, connecting with regions including the amygdala and striatum (Fig 2a).^71^, ^78^, ^84^ It is involved in responding to emotionally significant internal and external stimuli.^85^ Alterations in functional connectivity within the SN are seen in AD, and the dynamic interaction between the SN and DMN may be responsible for some of the neuropsychiatric symptoms seen in AD.^86^ For example, altered connectivity in the SN in early AD may be associated with increased response to emotionally salient stimuli and heightened emotional contagion; however, in some patients this may lead to agitation, irritability, and anxiety.^71^ Therefore pathology in cortical brain regions and aberrant connectivity within and across networks including the DMN, CEN, and SN lead to patterns of deficits in AD^78^ (Figure 2). The specific patterns of these deficits, including those involving awareness, may be heterogeneous as the location, extent, and chronological order of pathology may differ between patients.^78^, ^79^, ^87^ Similarly, different disorders of arousal in AD relate to variations in pathology in cortical, subcortical, and brainstem regions.^69^ Dysfunction and degeneration of the hypothalamic suprachiasmatic nucleus (SCN) is associated with circadian dysfunction in AD.^67^ Maintenance of arousal is associated with activity in the ascending reticular activating system (ARAS), which includes brainstem nuclei. Tau pathology appears early in brainstem nuclei in AD,^88^ and the combination of reduced activity in the ARAS and impaired descending cortical outputs results in difficulties maintaining arousal in severe AD.^70^

Neurodegeneration in AD is progressive, leading to dysfunction and disconnection in cortical networks.^89^ The behavioral evidence suggests this is associated with dysfunction in components and levels of awareness with worsening disease progression. However, almost all of the studies relating changes in awareness to neurodegeneration are from people with only mild–moderate AD. There has been a striking lack of research in people with severe dementia, where deficits in consciousness are potentially most profound and where the extent and nature of remaining awareness remains unclear. Although evidence links networks such as the DMN and CEN with elements of higher conscious experience, it is simplistic to attempt to map awareness directly onto brain networks, as fundamental questions remain regarding the mechanisms by which stimuli are consciously experienced at all. In other words, what is the minimal set of neuronal and computational mechanisms sufficient for a specific conscious perception (termed the NCCs^90^) and how do such physical mechanisms give rise to subjective experience? Not only is this the central question in consciousness science, it is particularly pertinent for people with AD, whose contents of awareness are eroded with progressive neurodegeneration. It appears that in severe AD, there is preservation of simple sensory perception; however, the NCCs of even simple conscious perception remain a subject of intense research. Current neurocomputational models of consciousness may offer potential explanations for how impairments in consciousness, at the levels of both arousal and awareness, occur in AD. Reciprocally, AD may act as a complex but consistent “lesion study” within which to test predictions made by these theories of consciousness.

## 4 NEURAL CORRELATES OF CONSCIOUSNESS AND AD

Central to questions of consciousness are the neural requirements or thresholds for a stimulus to enter awareness. One predominant model of consciousness, the global neuronal workspace theory (GNWT),^91^ predicts that for a stimulus to be consciously experienced it requires amplification of relevant sensory activity, long distance cortico‐cortical synchronization at beta and gamma frequencies, and ignition of a large‐scale prefronto‐parietal network.^92^ These neural mechanisms provide a global workspace for information to be maintained and accessed by a wide audience of brain networks, enabling conscious processing. In contrast, pre‐conscious stimuli are associated with increased activity in primary cortices but no ignition of widespread fronto‐parietal activation, and subliminal stimuli are not consciously experienced due to lack of activation in primary cortices.^93^, ^94^, ^95^ The theory predicts that loss of consciousness in conditions such as coma is associated with decreased activity in fronto‐parietal networks.^33^ Within AD, deficits in conscious perceptual awareness, even at the lowest level, could be due to a breakdown in any or all of these neural processes. Damage to critical brain regions and networks, including fronto‐parietal deficits, could prevent information from reaching or being maintained in a global workspace, which could be reduced or distorted by underlying pathology. A distorted, unreliable global workspace could result in the deficits and variability in conscious awareness seen in AD. AD pathology also disrupts the complex electrophysiological dynamics that may be required to maintain conscious mental representations.^78^

The integrated information theory (IIT) of consciousness^96^ predicts that consciousness is not necessarily related to function in specific brain regions, but is directly related to the ability of the brain to simultaneously integrate and differentiate information. This dynamic complexity can be quantified mathematically to provide objective markers of consciousness at an individual level in people under anesthesia, during sleep, and in coma.^97^ Within AD, dysfunction of brain networks may therefore prevent or distort the ability to maintain information in a global workspace and reduce the dynamic complexity of the brain, leading to the observed deficits in awareness and arousal. The development of biomarkers of consciousness from GNWT and IIT now provides opportunities to objectively test hypotheses of how consciousness becomes impaired in AD.

### 4.1 Biomarkers for assessing conscious awareness in severe AD

The lack of experimental data on conscious awareness in people with severe AD could be addressed using experimental paradigms that have successfully quantified awareness in people in vegetative states. Behavioral protocols validated for use in disorders of consciousness, such as the Coma Recovery Scale‐Revised (CRS‐R),^98^ should be combined with electophysiological and neuroimaging approaches. These methods could address crucial questions regarding consciousness in people with severe dementia, including whether awareness diminishes to the extent that patients become functionally unconscious in a similar manner to people in vegetative states, or whether variability in awareness remains.

### 4.2 Electrophysiological markers

Electrophysiological data using event‐related potentials and intracranial recordings reveal that conscious perception is associated not only with activity in primary sensory cortices but with late (i.e., 300 ms) amplification of relevant sensory activity and ignition of a prefronto‐parietal network,^99^ in line with predictions made by GNWT. In a study of infants, pictures of either faces or control stimuli were presented, at durations which could be consciously seen (> 100 ms) or were subliminal (< 100 ms) within a series of masking patterns. Faces presented long enough to be consciously seen were associated with a distributed and long‐lasting pattern of cortical activity that started 300 ms after the face stimulus.^100^ Similar markers could assess conscious perceptual awareness in severe AD.

**FIGURE 3:**
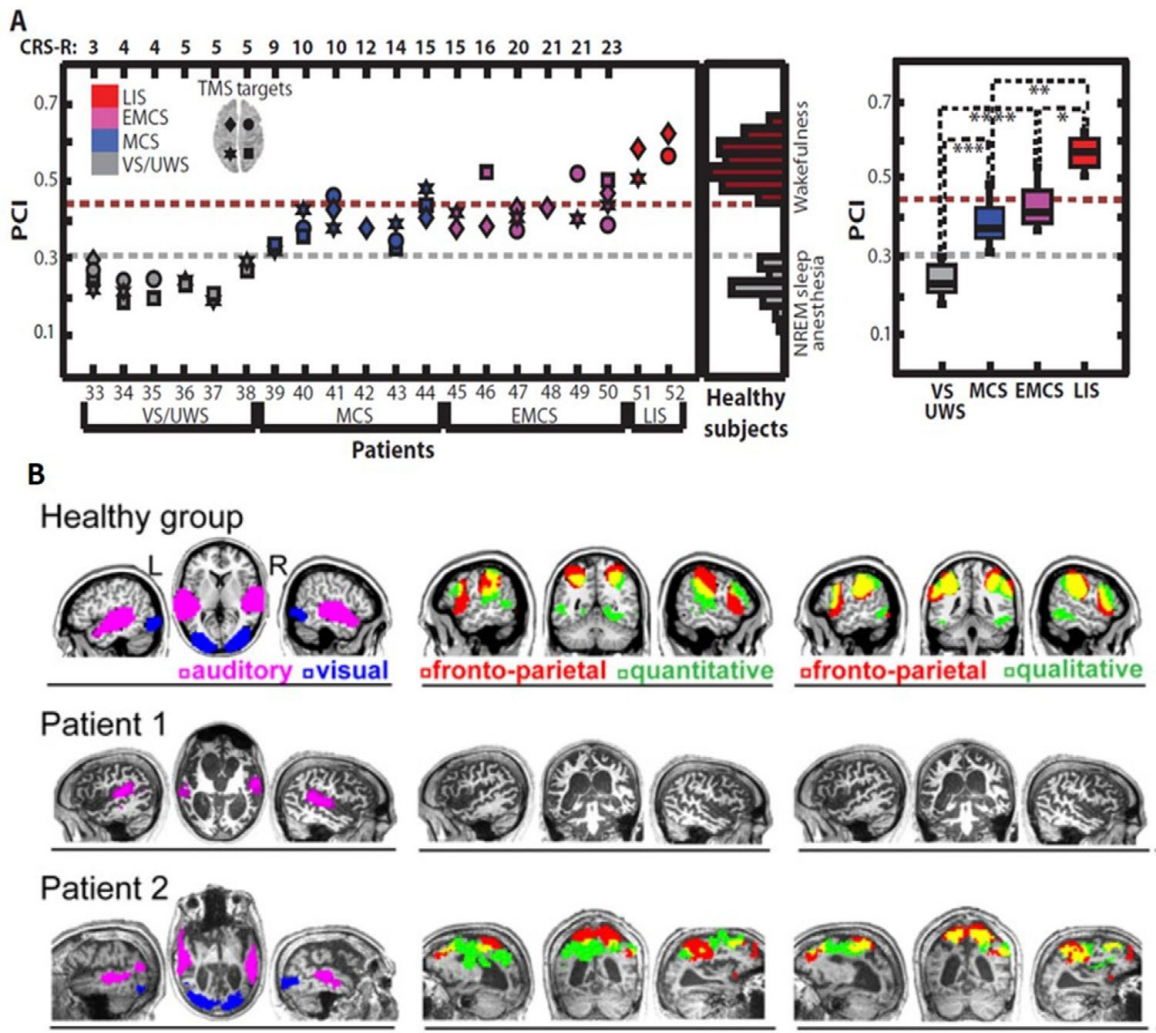
A, The Perturbational Complexity Index (PCI) as a marker of consciousness in different clinical conditions and states. Adapted from Casali et al.^97^ VS = vegetative state, MCS = minimally conscious state, EMCS = emerging from minimally conscious state. A key question is what is the range of consciousness as measured by PCI in people with severe Alzheimer's disease (AD)? B, Orthogonal activation in auditory, visual, and fronto‐parietal networks in response to watching a film. This has been used to demonstrate presence (Patient 2) or absence (Patient 1) of covert awareness in minimally conscious states and could be used similarly in people with severe AD. Adapted from Naci et al.^101^

A combination of transcranial magnetic stimulation (TMS) and electroencephalography (EEG) has been used to calculate markers of dynamical complexity, such as the Perturbational Complexity Index (PCI), to quantify consciousness in sleep, anesthesia, and patients in minimally conscious or vegetative states^97^ (Figure 3A). This TMS‐EEG method does not depend on the integrity of sensory and motor pathways, or require language comprehension or active participation and therefore offers an ideal tool to assess brain function in patients with severe dementia.

### 4.3 Neuroimaging markers

Passive neuroimaging paradigms enable the investigation of conscious experience in clinical populations who cannot provide verbal or behavioral report, such as those in vegetative states. For example, watching a short, attention‐engaging film leads to independent patterns of brain activity across auditory, visual, and fronto‐parietal networks. The time series of fronto‐parietal activity during such passive viewing demonstrates a shared pattern of experience across individuals and has been used to infer the presence of covert consciousness in behaviorally unresponsive patients^101^ (Figure 3B). This approach could assess consciousness in people with advanced AD by investigating whether, in response to visual and auditory stimuli, there is evidence of brain activity that goes beyond primary sensory cortices to activate a fronto‐parietal network, potentially reflecting higher‐level awareness.

## 5 THE IMPORTANCE OF AD AS A DISORDER OF CONSCIOUSNESS

Consideration of the effects of AD on the contents of consciousness has more than theoretical importance. It touches directly on the core experience of being human, and on what is most central and distressing for people with AD and their caregivers. There are clear clinical implications of the impairments in higher and lower levels of awareness that occur in AD. Anosognosia and impaired self‐awareness worsen treatment outcomes^102^ and medication management,^103^ and are associated with increased caregiver burden and burnout, social isolation, need for social services input, and institutionalization.^104^ Loss of awareness of socio‐emotional skills is related to behavioral and psychiatric disturbances,^46^ and deficits in reality monitoring may underpin delusions seen in AD.^27^, ^32^ Uncertainty remains as to the extent and nature of awareness in people with severe AD,^3^ with anecdotal reports describing lucid moments of unexpected higher awareness in response to specific stimulation such as music.^3^ However, it is not clear to what extent this reflects covert conscious experience or fluctuations in higher levels of awareness. Of crucial importance for the well‐being of people with AD and for health‐care services is to understand individual heterogeneity in awareness, and whether the expression of awareness may be dependent on, or modified by, external factors including the application of more need‐sensitive care.^12^, ^105^ There is a current drive to enrich environments in care homes to stimulate patients, but no clear evidence base as to how this should be done to improve the experience and outcomes of people with severe AD. A significant concern is that a caregiver may assume an uncommunicative person with severe AD is not consciously aware, leading to reduced interaction, which in turn reinforces neglectful care and negatively impacts both the person with dementia and their caregivers. In contrast, improving care workers’ ability to assess and understand awareness could positively reinforce engagement and improve patient‐centered care and quality of life in people with severe dementia.^105^ In other patient groups, such as those in vegetative states,^106^ the objective measurement of consciousness has provided a window into the lived experiences of these individuals, and significant improvements in their quality of life and outcomes have resulted.^107^ Using analogous markers could similarly improve understanding of the nature of consciousness in severe AD; enable professional caregivers and clinicians to more realistically view and respond to the needs of patients; and help develop and assess targeted interventions that improve awareness, quality of life, and care. Although this review focuses on AD, alterations of arousal and awareness also characterise other subtypes of dementia. For example, one of the core diagnostic criteria for dementia with Lewy bodies is fluctuating arousal,^108^ and behavioral variant frontotemporal dementia commonly presents with deficits in self‐awareness and empathy.^109^ This highlights the clinical and scientific need to clarify how different subtypes of dementia may lead to different impairments of consciousness, and whether, independent of etiology, all dementias could be considered disorders of consciousness, particularly at the more advanced stages.

**BOX 3 FUTURE DIRECTIONS**

**AD and consciousness science**

- What is the range of consciousness biomarkers seen in people with severe AD, as measured using markers of dynamical complexity, for example PCI?
- Do EEG, event‐related potential, and functional magnetic resonance imaging markers of conscious perceptual awareness demonstrate reduced fronto‐parietal “ignition” or sensory activation in moderate and severe AD?
- How does impairment in higher awareness relate to decline in executive function, attention, and episodic memory in early AD?
- How are higher facets of awareness (e.g., global metacognition, social awareness, and self‐awareness) and different levels of awareness related conceptually, neurobiologically, and cognitively in AD?
- Do apparent moments of lucidity reported in people with severe AD (e.g., in response to music) represent genuine fluctuations in consciousness?
- How do different subtypes of AD (e.g., PCA) or other common forms of dementia (e.g., behavioral variant fronto‐temporal dementia, dementia with Lewy bodies) differ in the impairment of awareness and how do the phenomenological differences relate to underlying pathology?
- Do different patterns of disruptions in the DMN, CEN, and salience networks map onto different patterns of impairments in the components of awareness?

**Management and care**

- Can interventions make people with AD more aware; for example music therapy, touch, or environmental enrichment?
- Can pharmacological approaches to enhance arousal and attention affect awareness in people with dementia?
- How might better knowledge of awareness in severe AD help caregivers and families understand and communicate with the person with dementia?
- How would a clearer picture of the trajectory of conscious awareness in AD impact n notions of personhood in dementia?
- Would improving anosognosia and self‐awareness in people with early dementia help them plan and accept care and reduce functional decline, or lead to increased depression and anxiety?
- Can targeting implicit and procedural learning be a successful approach for improving function in people with AD?

Considering AD as a disorder of consciousness leads to a number of important and testable questions for research and clinical care (see Box 3, Future Directions). Crucial to this discussion remains the imperative to maintain the dignity and humanity of people with severe AD, who may have significantly impaired consciousness. The personhood and wider social, interpersonal, cultural, and spiritual dimensions of a person with dementia remain of the utmost importance even if the underlying disease reduces their capacity for subjective conscious experience.^110^

## 6 CONCLUDING REMARKS

AD is characterized by dysfunction of arousal and awareness of the self and environment. It should be considered a disorder of consciousness, as this highlights the clinical characteristics of the disease and reflects what is important to patients and caregivers. Important clinical and research questions remain and there is an urgent need to understand and measure individual differences in awareness in people with AD, and how this can be modified by environmental stimuli, to ensure appropriate person‐centered care.

Recent development of novel, objective brain imaging biomarkers for consciousness offer an opportunity to apply these techniques to people with AD. The ability to track changes in markers of consciousness with disease progression and correlate this with changes in specific cognitive domains can aid our wider understanding of the NCC. In people with severe AD, it may also allow us to understand the level and nature of consciousness they experience and to focus interventions to improve care and quality of life.

## CONFLICTS OF INTEREST

The authors report no conflicts of interest
